# 

**Published:** 1904-01

**Authors:** 


### BOOKS RECEIVED.

Transactions of the State Medical Association of Texas. Thirty-fifth Annual Session held at San Antonio, Texas, April 28 to May 1, 1903. H. A. West, M. D., Secretary.

Fourth Annual Report of the Cancer Laboratory of the New York State Board of Health. Conducted at the Gratwick Research Laboratory, University of Buffalo. For the year 1902-3. Transmitted to the Legislature February 1, 1903. Albany: Evening Union Co. 1903.

A Reference Handbook of the Medical Sciences. Embracing the Entire Range of Scientific and Practical Medicine and Allied Science. By various writers. A new edition, completely revised and rewritten. Edited by Albert H. Buck, M. D., New York City. Nine volumes, imperial 8vo. Volume VII. Illustrated by chromolithographs and 688 half-tone and wood engravings. New York: William Wood & Co. 1902. (Price, muslin, $6.00 per volume; leather, $7.00 per volume; half morocco, $8.00 per volume.)

Progressive Medicine. Fifth Annual Series. Volume IV. December, 1903. A Quarterly Digest of Advances, Discoveries and Improvements in the Medical and Surgical Sciences. Edited by Hobart Amory Hare, M. D., Professor of Therapeutics and Material Medica in the Jefferson Medical College of Philadelphia. Octavo, 444 pages; illustrated. Philadelphia and New York: Lea Brothers & Co. (Per volume, $2.50, by express prepaid. Per annum, in four cloth-bound volumes, $10.00.)

Lectures on Neurology and Neuriatry, Psychology and Psychiatry. After the Methods of the class-room, to the Author's Students, and Designed also for General Practitioners of Medicine and Surgery. By C. H. Hughes, M. D., President of Faculty and Professor of Neurology, Psychiatry and Electrotherapy Barnes Medical College, Saint Louis. Edited by Prof. Marc Ray Hughes, M. D. Octavo, pages 417. Illustrated. Saint Louis: Hughes & Co. 1903. (Price, $3.00.)

A Non-Surgical Treatise on Diseases of the Prostate Gland and Adnexa. By George Whitfied Overall, M. D., Formerly Professor of Physiology in the Memphis Hospital Medical College. Duodecimo, pages 217. Illustrated. Chicago: Marsh & Grant Co. 1903.

A Compend of Pathology, General and Special. A Student's Manual in one volume. By Alfred Edward Thayer, M. D., Professor of Pathology, University of Texas. Duodecimo, pages 711; 131 illustrations. Second edition. Philadelphia: P. Blakiston's Son & Co. 1903.

Transactions of the American Surgical Association. Volume XXI. Edited by Richard H. Harte, M. D., Recorder of the Association. Philadelphia: Wm. J. Dornan, Printer. 1903.

Blakiston's Quiz Compends. Compend of Diseases of the Nose, Throat and Ear. By John Johnson Kyle, B. S., M. D., Lecturer on Otology, Rhinology and Laryngology in the Medical College of Indiana, Indianapolis. Pages, 280, with 85 illustrations. Philadelphia: P. Blakiston's Son & Co. 1903. (Price, 80 cents.)

Blood Pressure in Surgery. An Experimental and Clinical Research. The Cartwright Prize Essay for 1903. By George W. Crile, A. M., M. D., Professor of Clinical Surgery, Western Reserve Medical College, Cleveland. Octavo, pages 422. Illustrated. Philadelphia and London: J. B. Lippincott Co. 1903.

Mt. Sinai Hospital Reports. Volume III. For 1901 and 1902. Edited for the Medical Board by N. E. Brill, M. D. New York: Stettiner Bros. 1903.

Bovinine. A Compend of the Practice of Hematherapy as applied to General Medicine and Surgery. Octavo, pages 574. Illustrated. New York: The Bovinine Co. 1903.

